# A Unified Gas-Kinetic Particle Method for Radiation Transport in an Anisotropic Scattering Medium

**DOI:** 10.3390/e26010052

**Published:** 2024-01-06

**Authors:** Yuan Hu, Chang Liu, Huayun Shen, Gang Xiao, Jinghong Li

**Affiliations:** Institute of Applied Physics and Computational Mathematics, Beijing 100094, China; yuanhu@pku.org.cn (Y.H.); shen_huayun@iapcm.ac.cn (H.S.); xiaogang@zzhean.com (G.X.); li_jinghong@iapcm.ac.cn (J.L.)

**Keywords:** radiative transfer system, anisotropic scattering, asymptotic preserving, scattering phase function

## Abstract

In this paper, a unified gas kinetic particle (UGKP) method is developed for radiative transfer in both absorbing and anisotropic scattering media. This numerical method is constructed based on our theoretical work on the model reduction for an anisotropic scattering system. The macroscopic solver of this method directly solves the macroscopic anisotropic diffusion equations, eliminating the need to solve higher-order moment equations. The reconstruction of macroscopic scattering source in the microscopic solver, based on the multiscale equivalent phase function we proposed in this work, has also been simplified as one single scattering process, significantly reducing the computational costs. The proposed method has also the property of asymptotic preserving. In the optically thick regime, the proposed method solves the diffusion limit equations for an anisotropic system. In the optically thin regime, the kinetic processes of photon transport are simulated. The consistency and efficiency of the proposed method have been validated by numerical tests in a wide range of flow regimes. The novel equivalent scattering source reconstruction can be used for various transport processes, and the proposed numerical scheme is widely applicable in high-energy density engineering applications.

## 1. Introduction

Thermal radiative transfer (TRT) equations, which describe the time evolution of radiative intensity and its interaction with the background material, have wide applications in astrophysics, atmospheric physics, inertial confinement fusion (ICF), high-temperature flow systems, plasma physics, etc. [1,2,3,4,5,6,7,8]. TRT equations are intrinsically nonlinear due to the absorption–emission process, which renders the system difficult to solve [9,10,11]. Furthermore, the intrinsic high dimensionality of TRT equations significantly increases the computational costs. Developing numerical methods with high accuracy and high efficiency has become an important topic over the past few decades.

Currently, three kinds of models are used to describe the radiation transport process: the kinetic model, the macroscopic moment model, and the machine learning model. The kinetic model, also known as the Boltzmann transport equation, comprehensively describes the photon transport, absorbing, emission, and scattering processes at a mesoscale level. It is the most fundamental equation of photon transport, with seven dimensions, i.e., the physical space, the velocity space, the frequency space, and time. However, it is computationally expensive to solve directly. The macroscopic moment model, in contrast, offers a means to reduce the high-dimensional Boltzmann transport equation to a low-dimensional equation based on the spherical harmonic function expansion [12]. Nonetheless, further research is necessary to bolster the reliability of its closed function. The machine learning model, as a relatively new model, utilizes deep neural networks to develop efficient macroscopic models [13]. However, the generalization ability and confidence of the macroscopic models generated by deep neural networks require additional validation.

Generally, the numerical methods for kinetic equations are categorized into the deterministic method and the stochastic method. The deterministic methods include the macroscopic moments method [14,15], and microscopic discrete ordinate (SN) method [16,17,18,19,20,21]. The moment method expands the radiant intensity in a spherical harmonic function space, and the SN method discretizes the velocity space into grid points. For the stochastic method, the radiative transfer equation is solved by simulating the interactions of individual radiation particles with the background material. The implicit Monte Carlo (IMC) method, proposed by Fleck and Cummings [22], is a popular Monte Carlo method for solving the TRT equations. The deterministic method has an explicit formation, which could provide analytic solutions. However, it will suffer from the nonphysical ray effects. Conversely, the stochastic method does not suffer the ray effects and can be applied for high-dimensional phase space directly with good robustness. However, the stochastic process of individual particles will lead to statistical results. In addition, both the deterministic method and stochastic method are single-scale numerical methods, which are unsuitable for problems with complex regimes.

Recently, the multiscaled unified gas kinetic scheme (UGKS) method has been developed to address problems spanning optically thin and thick regimes [23,24,25,26]. The UGKS method utilizes a finite volume formulation to solve the macroscopic energy equations, while the discrete ordinates method (DOM) is employed for solving the microscopic transport equation [27,28]. The integral solution of the transport equation bridges the macroscopic and microscopic equations, which covers the physics from the free transport to the diffusion limit and makes the UGKS method asymptotic preserving (AP). Based on the framework of UGKS, different numerical schemes have been constructed, including the discrete unified gas kinetic scheme (DUGKS) method [29,30,31], the implicit unified gas kinetic scheme (IUGKS) method [32,33,34,35], the unified gas-kinetic particle (UGKP) method [36,37], and the unified gas-kinetic wave-particle (UGKWP) method [38,39,40]. These methods share a similar framework with the UGKS method, making them asymptotic preserving as well. Furthermore, the Monte Carlo solver of the UGKP and UGKWP methods avoids the interdependence of complex unstructured mesh in microscopic transport equation solving, which makes the UGKP and UGKWP methods more suitable for problems with such mesh than the UGKS method.

In our prior work, we extended the UGKP method for the frequency-dependent radiative system, considering both absorption–emission and isotropic scattering processes under a general unstructured mesh [37]. However, practical applications usually involve media with anisotropic scattering properties. Up until this point, both the UGKP and UGKWP methods have not considered the effects of the anisotropic scattering process yet. In the present work, we take the next step by extending the UGKP method for the anisotropic scattering system, building upon our previous theoretical foundations [41]. The rest of this paper is organized as follows. In Section 2.1, we briefly introduce the UGKP method for the isotropic scattering system (hereinafter referred to as the isotropic-UGKP method). Then, we present the UGKP method for the anisotropic scattering system (hereinafter referred to as the anisotropic-UGKP method) in Section 2.2, based on the foundation laid out in Section 2.1. The asymptotic preserving property of the proposed anisotropic-UGKP method will be discussed in the following Section 2.3. The results of several numerical results will be presented in Section 3 to validate the proposed anisotropic-UGKP method. Finally, we conclude and discuss future work in Section 4.

## 2. Numerical Methods

In this section, we present our proposed UGKP method for a gray radiative transfer system with an anisotropic scattering process. The following formulas are presented in a two-dimensional Cartesian space. The angle direction is denoted by $\vec{\Omega }=\left(\mu ,\xi \right)$ with $\mu =\sqrt{1-\zeta^{2}}\cos\gamma$ and $\xi =\sqrt{1-\zeta^{2}}\sin\gamma$ , where $\zeta \in \left[-1,1\right]$ is the cosine value of the angle between the propagation direction $\vec{\Omega }$ and the *z*-axis, and $\gamma \in \left[0,2\pi \right)$ is the angle between the projection vector of $\vec{\Omega }$ onto the xy -plane and the *x*-axis. Because of the symmetry of angular distribution in the two-dimensional Cartesian case, we only need to consider ζ>0 .

### 2.1. Isotropic-UGKP Method

In this subsection, we provide a brief introduction to the isotropic-UGKP method for the gray radiative transfer system.

#### 2.1.1. Gray Radiative Transfer System with Isotropic Scattering

The gray radiative transfer equations describe the transport of radiation and its energy exchange with material. Under the assumption of the local thermal equilibrium (LTE), the time-dependent gray radiative transfer equations in the absence of both external and internal sources can be written in the following scaled form:

(1) $$\left\{\begin{array}{cc} \; & \frac{\varepsilon }{c}\frac{\partial I}{\partial t}+\vec{\Omega }\cdot \nabla I=L_{a}^{\varepsilon }\sigma_{a}\left(\frac{1}{2\pi }\phi -I\right)+L_{s}^{\varepsilon }\sigma_{s}\left(\frac{1}{2\pi }\rho -I\right),\\ \; & C_{V}\frac{\partial T}{\partial t}\equiv \frac{\partial U_{m}}{\partial t}=\frac{L_{a}^{\varepsilon }\sigma_{a}}{\varepsilon }\int \left(I-\frac{1}{2\pi }\phi \right)d\vec{\Omega }. \end{array}\right.$$

where $I\left(t,\vec{r},\vec{\Omega }\right)$ is the radiation intensity, which depends on time *t*, spatial variable $\vec{r}$ , and angular variable $\vec{\Omega }$ ; $T\left(t,\vec{r}\right)$ is the material temperature; $\phi =acT^{4}$ with the radiation constant *a* and the speed of light *c* and $\rho =\int Id\vec{\Omega }$ ; $\sigma_{a}\left(\vec{r},T\right)$ is the absorption coefficient; $\sigma_{s}\left(\vec{r},T\right)$ is the scattering coefficient; ε>0 is the Knudsen number; $L_{a}^{\varepsilon }$ and $L_{s}^{\varepsilon }$ are two parameters depending on ε ; $U_{m}\left(\vec{r},t\right)$ is the material energy density; and $C_{V}>0$ is the heat capacity. Taking the angular integration of the radiation transport equation in (1), we obtain the following macroscopic system:

(2) $$\left\{\begin{array}{cc} \; & \frac{\varepsilon }{c}\frac{\partial \rho }{\partial t}+\nabla \cdot \langle \vec{\Omega }I\rangle =L_{a}^{\varepsilon }\sigma_{a}\left(\phi -\rho \right),\\ \; & \frac{\partial \phi }{\partial t}=\frac{L_{a}^{\varepsilon }\sigma_{a}}{\varepsilon }\beta \left(\rho -\phi \right), \end{array}\right.$$

where $\langle \vec{\Omega }I\rangle =\int \vec{\Omega }Id\vec{\Omega }$ , and $\beta =\partial \phi /\partial U_{m}=4acT^{3}/C_{v}$ .

#### 2.1.2. Macroscopic Solver for the Isotropic-UGKP Method

**Figure 1:**
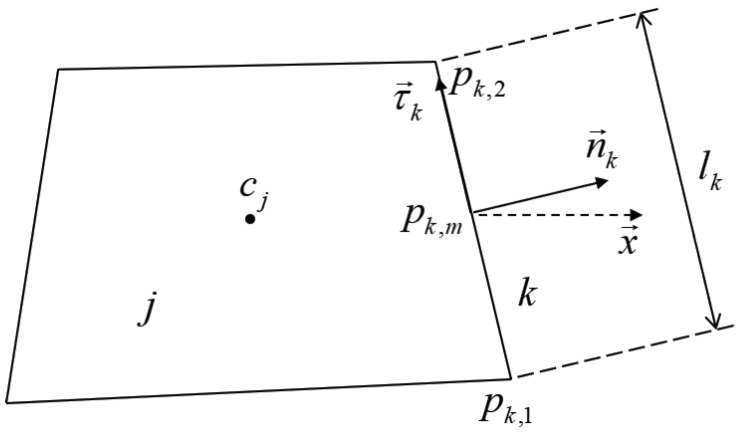
A cell *j* of the generalized quadrilateral mesh with $c_{j}$ as the cell center and $p_{k,m}$ as the center of edge *k*. The length of the edge *k* is $l_{k}$ . The two vertexes of edge *k* are ${\vec{p}}_{k,1}$ and ${\vec{p}}_{k,2}$ , and the unit normal and tangential vector are ${\vec{n}}_{k}$ and ${\vec{\tau }}_{k}$ , respectively [37].

Equation (2) can be discretized on both spatial and time variables based on the finite volume method. On the unstructured mesh illustrated in Figure 1, a conservative finite volume numerical scheme for the macroscopic system (2) has the following form:

(3) $$\left\{\begin{array}{cc} \; & \rho_{j}^{n+1}=\rho_{j}^{n}-\frac{\Delta t}{V_{j}}\sum_{k}\Phi_{j,k}^{n+1}+\frac{c\Delta tL_{a}^{\varepsilon }{\left(\sigma_{a}\right)}_{j}^{n+1}}{\varepsilon }\left(\phi_{j}^{n+1}-\rho_{j}^{n+1}\right),\\ \; & \phi_{j}^{n+1}=\phi_{j}^{n}+\frac{\Delta tL_{a}^{\varepsilon }{\left(\sigma_{a}\beta \right)}_{j}^{n+1}}{\varepsilon }\left(\rho_{j}^{n+1}-\phi_{j}^{n+1}\right), \end{array}\right.$$

where $\Delta t=t_{n+1}-t_{n}$ is the time interval; $\rho_{j}^{n+1}$ and $\phi_{j}^{n+1}$ are the cell averaged value at time $t_{n+1}$ in cell *j*; and $\Phi_{j,k}^{n+1}$ is the macroscopic flux across the cell edge *k*. The $\rho_{j}^{n+1}$ , $\phi_{j}^{n+1}$ , and $\Phi_{j,k}^{n+1}$ are defined as:

(4) $$\begin{array}{cc} \; & \rho_{j}^{n+1}=\frac{1}{V_{j}}\int_{V_{j}}\rho \left(t_{n+1},x,y\right)dxdy,\\ \; & \phi_{j}^{n+1}=\frac{1}{V_{j}}\int_{V_{j}}\phi \left(t_{n+1},x,y\right)dxdy,\\ \; & \Phi_{j,k}^{n+1}=\frac{cl_{k}}{\varepsilon \Delta t}\int_{t_{n}}^{t_{n+1}}{\vec{n}}_{k}\cdot \langle \vec{\Omega }I\rangle \left(t,p_{k,m}\right)dt, \end{array}$$

where $V_{j}$ is the volume of mesh *j*; $p_{k,m}$ is the center of edge *k*; $l_{k}$ is the length of edge *k*; and ${\vec{n}}_{k}$ is the unit normal vector of edge *k*. The construction of $\Phi_{j,k}^{n+1}$ is the key to the isotropic-UGKP method.

To calculate the macroscopic flux $\Phi_{j,k}^{n+1}$ in Equation (4), one must determine the radiation intensity in the vicinity of the center of edge *k* by solving the following equation:

(5) $$\left\{\begin{array}{cc} \; & \frac{\varepsilon }{c}\frac{\partial I}{\partial t}+\mu^{\prime }\frac{\partial I}{\partial x^{\prime }}+\xi^{\prime }\frac{\partial I}{\partial y^{\prime }}=L_{a}^{\varepsilon }\sigma_{a}\left(\frac{1}{2\pi }\phi -I\right)+L_{s}^{\varepsilon }\sigma_{s}\left(\frac{1}{2\pi }\rho -I\right),\\ \; & {\left.I\left(x^{\prime },y^{\prime },t\right)\right|}_{t=t_{n}}=I\left(x^{\prime },y^{\prime },t_{n}\right). \end{array}\right.$$

Equation (5) is formatted under the local coordinate system on edge center $p_{k,m}$ , with $x^{\prime }$ and $y^{\prime }$ as the local orthogonal coordinates, and $\mu^{\prime }$ and $\xi^{\prime }$ as the local normal and tangential directions. The integral solution of Equation (5) is:

(6) $$\begin{array}{cc} I\left(t,0,0\right)= & e^{-\lambda \left(t-t_{n}\right)}I\left(t_{n},-\frac{\mu^{\prime }c}{\varepsilon }\left(t-t_{n}\right),-\frac{\xi^{\prime }c}{\varepsilon }\left(t-t_{n}\right)\right)\\ \; & +\int_{t_{n}}^{t}e^{-\lambda \left(t-s\right)}\left[\begin{array}{c} \frac{cL_{a}^{\varepsilon }\sigma_{a}}{\varepsilon }\frac{1}{2\pi }\phi \left(s,-\frac{\mu^{\prime }c}{\varepsilon }\left(t-s\right),-\frac{\xi^{\prime }c}{\varepsilon }\left(t-s\right)\right)\\ +\frac{cL_{s}^{\varepsilon }\sigma_{s}}{\varepsilon }\frac{1}{2\pi }\rho \left(s,-\frac{\mu^{\prime }c}{\varepsilon }\left(t-s\right),-\frac{\xi^{\prime }c}{\varepsilon }\left(t-s\right)\right) \end{array}\right]ds, \end{array}$$

where $\lambda =c\left(L_{a}^{\varepsilon }\sigma_{a}+L_{s}^{\varepsilon }\sigma_{s}\right)/\varepsilon$ . Then, the macroscopic flux is evaluated by

(7) $$\Phi_{j,k}^{n+1}=\frac{cl_{k}}{\varepsilon \Delta t}\int_{t_{n}}^{t_{n+1}}\langle \mu^{\prime }I\rangle \left(t,0,0\right)dt.$$

The macroscopic quantities ϕ and ρ in the integral solution (6) can be reconstructed by a piecewise linear polynomial around the edge *k* as follows:

(8) $$\begin{array}{cc} \; & \phi \left(t,x^{\prime },y^{\prime }\right)=\phi_{j,k}^{n+1}+\delta_{t}\phi_{j,k}^{n+1}\left(t-t_{n+1}\right)+\delta_{y^{\prime }}\phi_{j,k}^{n+1}y^{\prime }+\delta_{x^{\prime }}\phi_{j,k}^{n+1}x^{\prime },\\ \; & \rho \left(t,x^{\prime },y^{\prime }\right)=\rho_{j,k}^{n+1}+\delta_{t}\rho_{j,k}^{n+1}\left(t-t_{n+1}\right)+\delta_{y^{\prime }}\rho_{j,k}^{n+1}y^{\prime }+\delta_{x^{\prime }}\rho_{j,k}^{n+1}x^{\prime }, \end{array}$$

with $\phi_{j,k}^{n+1}$ and $\rho_{j,k}^{n+1}$ as the value at edge *k*. The spatial derivatives in (8) are calculated by:

(9) $$\begin{array}{cc} \; & \delta_{x^{\prime }}\phi_{j,k}^{n+1}=\frac{\phi_{j^{\prime }}^{n+1}-\phi_{j}^{n+1}-\left(\tau_{j,k}^{-}+\tau_{j,k}^{+}\right)\left({\hat{\phi }}_{k,2}^{n+1}-{\hat{\phi }}_{k,1}^{n+1}\right)}{l_{j,k}^{-}+l_{j,k}^{+}},\;\delta_{y^{\prime }}\phi_{j,k}^{n+1}=\frac{{\hat{\phi }}_{k,2}^{n+1}-{\hat{\phi }}_{k,1}^{n+1}}{l_{k}},\\ \; & \delta_{x^{\prime }}\rho_{j,k}^{n+1}=\frac{\rho_{j^{\prime }}^{n+1}-\rho_{j}^{n+1}-\left(\tau_{j,k}^{-}+\tau_{j,k}^{+}\right)\left({\hat{\rho }}_{k,2}^{n+1}-{\hat{\rho }}_{k,1}^{n+1}\right)}{l_{j,k}^{-}+l_{j,k}^{+}},\;\delta_{y^{\prime }}\rho_{j,k}^{n+1}=\frac{{\hat{\rho }}_{k,2}^{n+1}-{\hat{\rho }}_{k,1}^{n+1}}{l_{k}}, \end{array}$$

where $j^{\prime }$ denotes the neighboring cell which has the common edge *k* with cell *j*. The ${\hat{\phi }}_{k,1}^{n+1}$ , ${\hat{\phi }}_{k,2}^{n+1}$ , ${\hat{\rho }}_{k,1}^{n+1}$ , and ${\hat{\rho }}_{k,2}^{n+1}$ , as the macroscopic quantities on the two vertexes ( $p_{k,1}$ and $p_{k,2}$ ) of edge *k*, are calculated as the average macroscopic quantities of those cells that shared the common vertex. The projected lengths in Equation (9) are given by:

(10) lj,k−=r→cj,pk,m·n→k,lj,k+=r→pk,m,cj′·n→k,τj,k−=r→cj,pk,m·τ→kr→cj,pk,m·τ→klklk,τj,k+=r→pk,m,cj′·τ→kr→pk,m,cj′·τ→klklk.

The time derivatives in (8) are given by:

(11) $$\delta_{t}\phi_{j,k}^{n+1}=\frac{\phi_{j,k}^{n+1}-\phi_{j,k}^{n}}{\Delta t},\;\;\delta_{t}\rho_{j,k}^{n+1}=\frac{\rho_{j,k}^{n+1}-\rho_{j,k}^{n}}{\Delta t}.$$

With the reconstruction of Equations (8)–(11), the macroscopic flux $\Phi_{j,k}^{n+1}$ is evaluated by:

(12) $$\begin{array}{cc} \; & \Phi_{j,k}^{n+1}=\Phi_{j,k}^{n+1,micro}+\Phi_{j,k}^{n+1,macro},\\ \; & \Phi_{j,k}^{n+1,micro}=\langle \frac{cl_{k}}{\varepsilon \Delta t}\int_{t_{n}}^{t_{n+1}}\mu^{\prime }e^{-\lambda \left(t-t_{n}\right)}I\left(t_{n},-\frac{\mu^{\prime }c}{\varepsilon }\left(t-t_{n}\right),-\frac{\xi^{\prime }c}{\varepsilon }\left(t-t_{n}\right)\right)dt\rangle ,\\ \; & \Phi_{j,k}^{n+1,macro}=\frac{2\pi }{3}{\left(D^{1}\right)}_{j,k}^{n+1}\delta_{x^{\prime }}\phi_{j,k}^{n+1}+\frac{2\pi }{3}{\left(D^{2}\right)}_{j,k}^{n+1}\delta_{x^{\prime }}\rho_{j,k}^{n+1}, \end{array}$$

with the effective diffusion coefficients in $\Phi_{j,k}^{n+1,macro}$ as:

(13) $$\left\{\begin{array}{cc} \; & D^{1}=-\frac{c^{3}l_{k}L_{a}^{\varepsilon }\sigma_{a}}{2\pi \Delta t\varepsilon^{3}\lambda^{2}}\left[\Delta t\left(1+e^{-\lambda \Delta t}\right)-\frac{2}{\lambda }\left(1-e^{-\lambda \Delta t}\right)\right],\\ \; & D^{2}=-\frac{c^{3}l_{k}L_{s}^{\varepsilon }\sigma_{s}}{2\pi \Delta t\varepsilon^{3}\lambda^{2}}\left[\Delta t\left(1+e^{-\lambda \Delta t}\right)-\frac{2}{\lambda }\left(1-e^{-\lambda \Delta t}\right)\right]. \end{array}\right.$$

The $\Phi_{j,k}^{n+1,micro}$ of the macroscopic flux (12) contributes from microscopic photons with free transport, while the $\Phi_{j,k}^{n+1,macro}$ accounts for the contribution from macroscopic diffusive flux. Therefore, the macroscopic flux (12) is a multiscale numerical flux, which bridges the microscopic radiant flux and macroscopic diffusive flux.

Upon calculating the $\Phi_{j,k}^{n+1,micro}$ in Equation (12) by the microscopic solver (which will be discussed in the next subsection), a coupled nonlinear system for the macroscopic quantities ϕ and ρ is established. This system is solved by the following source iteration Algorithm 1.
**Algorithm 1** Source iteration algorithm for macroscopic energy evolution.
1:Initialize flow field with $\phi_{j}^{n+1,0}=\phi_{j}^{n}$ and $\rho_{j}^{n+1,0}=\rho_{j}^{n}$
2:**while** Residuals of the macroscopic quantities $\phi_{j}^{n+1,s}$ and $\rho_{j}^{n+1,s}$ do not meet the convergence criterion **do**3:   Compute ${\left(\sigma_{a}\right)}_{j}^{n+1,s}$ , ${\left(\sigma_{s}\right)}_{j}^{n+1,s}$ , ${\left(D^{1}\right)}_{j,k}^{n+1,s}$ , and ${\left(D^{2}\right)}_{j,k}^{n+1,s}$ with $T_{j}^{n+1,s}=\sqrt[{4}]{\phi_{j}^{n+1,s}/ac}$ .4:   Solve the inner-loop linear system to update $\rho_{j}^{n+1,s+1}$ and $\phi_{j}^{n+1,s+1}$ with

(14)
$$\left\{\begin{array}{cc} \; & \rho_{j}^{n+1,s+1}=\rho_{j}^{n}-\frac{\Delta t}{V_{j}}\sum_{k}\Phi_{j,k}^{n+1,s}+\frac{c\Delta tL_{a}^{\varepsilon }{\left(\sigma_{a}\right)}_{j}^{n+1,s}}{\varepsilon }\left(\phi_{j}^{n+1,s+1}-\rho_{j}^{n+1,s+1}\right),\;\\ \; & \phi_{j}^{n+1,s+1}=\phi_{j}^{n}+\frac{\Delta tL_{a}^{\varepsilon }{\left(\sigma_{a}\beta \right)}_{j}^{n+1,s}}{\varepsilon }\left(\rho_{j}^{n+1,s+1}-\phi_{j}^{n+1,s+1}\right), \end{array}\right.$$

with $\Phi_{j,k}^{n+1,s}$ in (12).5:**end while**6:Update the macroscopic quantities $\rho_{j}^{n+1}=\rho_{j}^{n+1,s+1}$ , $\phi_{j}^{n+1}=\phi_{j}^{n+1,s+1}$ , and $T_{j}^{n+1}=\sqrt[{4}]{\phi_{j}^{n+1}/ac}$ .


#### 2.1.3. Microscopic Solver for the Isotropic-UGKP Method

Following the first-order expansion of the macroscopic quantities, Equation (6) can be written as:

(15) $$\begin{array}{cc} \; & \begin{array}{cc} I\left(t_{n+1},x^{\prime },y^{\prime }\right) & =e^{-\lambda \Delta t}I\left(t_{n},x^{\prime }-\frac{\mu^{\prime }c}{\varepsilon }\Delta t,y^{\prime }-\frac{\xi^{\prime }c}{\varepsilon }\Delta t\right)+\frac{1}{\lambda }\left(1-e^{-\lambda \Delta t}\right)\left[\frac{cL_{a}^{\varepsilon }\sigma_{a}}{\varepsilon }\frac{\phi_{j}^{n+1}}{2\pi }+\frac{cL_{s}^{\varepsilon }\sigma_{s}}{\varepsilon }\frac{\rho_{j}^{n+1}}{2\pi }\right]\\ \; & =e^{-\lambda \Delta t}I^{transport}\left(t_{n+1},x^{\prime },y^{\prime }\right)+\left(1-e^{-\lambda \Delta t}\right){\left(I^{diffusion}\right)}_{j}^{n+1}, \end{array}\\ \; & I^{transport}\left(t_{n+1},x^{\prime },y^{\prime }\right)=I\left(t_{n},x^{\prime }-\frac{\mu^{\prime }c}{\varepsilon }\Delta t,y^{\prime }-\frac{\xi^{\prime }c}{\varepsilon }\Delta t\right),\\ \; & {\left(I^{diffusion}\right)}_{j}^{n+1}=\frac{1}{\lambda }\left[\frac{cL_{a}^{\varepsilon }\sigma_{a}}{\varepsilon }\frac{\phi_{j}^{n+1}}{2\pi }+\frac{cL_{s}^{\varepsilon }\sigma_{s}}{\varepsilon }\frac{\rho_{j}^{n+1}}{2\pi }\right]. \end{array}$$

Equation (15) means that the radiation intensity at $t_{n+1}$ is a combination of the free transport and diffusion radiation intensity whose relative contribution is determined by the $e^{-\lambda \Delta t}$ and $1-e^{-\lambda \Delta t}$ term. With the macroscopic quantities $\rho_{j}^{n+1}$ and $\phi_{j}^{n+1}$ acquired through the macroscopic solver in Algorithm 1, the microscopic radiation intensity at $t_{n+1}$ can be recovered based on Equation (15). The $e^{-\lambda \Delta t}I^{transport}\left(t_{n+1},x^{\prime },y^{\prime }\right)$ in (15) contributes from freely streamed photons in the time step $t\in \left[t_{n},t_{n+1}\right]$ , which can be further tracked in the next time step. Thus, the re-emitted and scattered photons are re-sampled from $\left(1-e^{-\lambda \Delta t}\right){\left(I^{diffusion}\right)}_{j}^{n+1}$ in (15) only. The numbers of the re-sampled photons are determined by:

(16) $$\begin{array}{cc} \; & E_{j,re-emitted}=\left(1-e^{-\lambda \Delta t}\right)\frac{1}{\lambda }\frac{cL_{a}^{\varepsilon }\sigma_{a}}{\varepsilon }\phi_{j}^{n+1},\;N_{j,re-emitted}=\frac{E_{j,re-emitted}}{w_{ref}},\\ \; & E_{j,scattered}=\left(1-e^{-\lambda \Delta t}\right)\frac{1}{\lambda }\frac{cL_{s}^{\varepsilon }\sigma_{s}}{\varepsilon }\rho_{j}^{n+1},\;N_{j,scattered}=\frac{E_{j,scattered}}{w_{ref}}, \end{array}$$

where $E_{j,re-emitted}$ and $E_{j,scattered}$ are the total energy of re-emitted and scattered photons of cell *j*, respectively; and $w_{ref}$ is the reference weight of photons. Then, the re-emitted and scattered photons are sampled in each mesh isotropically (pointed out by the 1/2π in Equation (15)) and uniformly.

The photon-tracking process in the isotropic-UGKP method is similar to the traditional Monte Carlo method, except that those “interacted” photons will be immediately removed from the simulation. The subsequent behavior of these “interacted” photons is evaluated by the macroscopic solver in Algorithm 1 instead of the meticulous tracking involved in the traditional Monte Carlo method. During particle tracking, the $\Phi_{j,k}^{n+1,micro}$ in Equation (12) is calculated by:

(17) $$\Phi_{j,k}^{n+1,micro}=\sum_{i}1\left(\mu^{\prime }\right)w_{i},\;1\left(\mu^{\prime }\right)=\left\{\begin{array}{c} 1,\mu^{\prime }>0\\ -1,\mu^{\prime }<0 \end{array}\right.,$$

where $\sum_{i}$ stands for the summation of all photons *i* that are transported across the edge *k*, and $w_{i}$ is the weight of MC particle *i*.

The microscopic solver of the isotropic-UGKP method is summarized by the following Algorithm 2.
**Algorithm 2** Photon re-sampling and tracking algorithm for microscopic radiation intensity evolution.
1:Photon re-sampling:2:**for all** mesh cell **do**3:   Re-sample the re-emitted and scattered photons isotropically and uniformly in mesh cell.4:**end for**5: 6:Photon tracking:7:**for all** photon **do**8:   **repeat**9:     Track photon and calculate the $\Phi_{j,k}^{n+1,micro}$ by (17).10:  **until** Photon leaks out of the system **or** Photon has “interacted” with material **or** Photon reaches the end of time step11:  **if** Photon leaks out of the system **or** Photon has “interacted” with material **then**12:    Remove the photon from the simulation.13:  **else if** Photon reaches the end of time step **then**14:    Continually simulate the photon in the next time step.15:  **end if**16:**end for**


### 2.2. Anisotropic-UGKP Method

In this subsection, we present the anisotropic-UGKP method for the gray radiative transfer system. We begin by highlighting the distinction between the isotropic and anisotropic systems and subsequently discuss the modifications made to transition from the isotropic-UGKP method to the anisotropic-UGKP method.

#### 2.2.1. The Difference between Isotropic and Anisotropic System

For an anisotropic system, the scaled form of the time-dependent gray radiative transfer equations under the same condition in Section 2.1.1 are:

(18) $$\left\{\begin{array}{cc} \; & \frac{\varepsilon }{c}\frac{\partial I}{\partial t}+\vec{\Omega }\cdot \nabla I=L_{a}^{\varepsilon }\sigma_{a}\left(\frac{1}{2\pi }\phi -I\right)+L_{s}^{\varepsilon }\sigma_{s}\left(S-I\right),\\ \; & C_{V}\frac{\partial T}{\partial t}\equiv \frac{\partial U_{m}}{\partial t}=\frac{L_{a}^{\varepsilon }\sigma_{a}}{\varepsilon }\int \left(I-\frac{1}{2\pi }\phi \right)d\vec{\Omega }, \end{array}\right.$$

where $S=\int p\left({\vec{\Omega }}^{\prime }\to \vec{\Omega }\right)I\left({\vec{\Omega }}^{\prime }\right)d{\vec{\Omega }}^{\prime }$ represents the scattering source of anisotropic scattering. The $p\left({\vec{\Omega }}^{\prime }\to \vec{\Omega }\right)$ here is the scattering phase function, which depends on the incoming direction ${\vec{\Omega }}^{\prime }$ and the outgoing direction $\vec{\Omega }$ . Compared with (1) and (18), the only difference between isotropic and anisotropic scattering systems is the scattering source term, changing from ρ/2π into *S*.

As previously discussed in our theoretical work [41], the scattering phase function can be approximated by a finite series of polynomials:

(19) $$p\left({\vec{\Omega }}^{\prime }\to \vec{\Omega }\right)=\sum_{l=0}^{L}C_{l}^{*}{\left({\vec{\Omega }}^{\prime }\cdot \vec{\Omega }\right)}^{l}=\sum_{l=0}^{L}C_{l}^{*}{\left(cos\theta \right)}^{l},$$

with $C_{l}^{*}$ as the corresponding coefficients of polynomial ${\left({\vec{\Omega }}^{\prime }\cdot \vec{\Omega }\right)}^{l}$ , and θ representing the scattering angle between ${\vec{\Omega }}^{\prime }$ and $\vec{\Omega }$ . Based on (19), the scattering source *S* maintains a relationship with the moments of radiation intensity *I* through:

(20) $$S=\sum_{l=0}^{L}C_{l}^{*}\sum_{i_{1}i_{2}\cdots i_{l}}\left(\Omega_{i_{1}i_{2}\cdots i_{l}}^{\left(l\right)}J_{i_{1}i_{2}\cdots i_{l}}^{\left(l\right)}\right),$$

where $\Omega_{i_{1}i_{2}\cdots i_{l}}^{\left(l\right)}=\Omega_{i_{1}}\Omega_{i_{2}}\cdots \Omega_{i_{l}}$ , the index $i_{k}\in \left\{x,y,z\right\}$ with the corresponding direction cosine $\Omega_{i_{k}}\in \left\{\mu ,\xi ,\zeta \right\}$ and $k\in \left[0,l\right]$ ; $J^{\left(l\right)}$ is the *l*th-order moments of *I*, and $J_{i_{1}i_{2}\cdots i_{l}}^{\left(l\right)}$ is the $\left\{i_{1}i_{2}\cdots i_{l}\right\}$ component of $J^{\left(l\right)}$ ; $\sum_{i_{1}i_{2}\cdots i_{l}}$ encompasses all possible combinations of index $\left\{i_{1}i_{2}\cdots i_{l}\right\}$ ; and $\Omega_{i_{1}i_{2}\cdots i_{l}}^{\left(l\right)}=\Omega_{i_{1}}\Omega_{i_{2}}\cdots \Omega_{i_{l}}$ shares the same index $\left\{i_{1}i_{2}\cdots i_{l}\right\}$ as $J_{i_{1}i_{2}\cdots i_{l}}^{\left(l\right)}$ . The necessary modifications of the macroscopic and microscopic solver for the anisotropic-UGKP method, because of the scattering source changing from ρ/2π in an isotropic system to *S* in an anisotropic system, are discussed in the following subsections.

#### 2.2.2. Macroscopic Solver for the Anisotropic-UGKP Method

In our prior work, we proved that the pure anisotropic scattering radiative transfer equation satisfies the diffusion equation in the optically thick limiting regime [41]. The diffusion coefficient of pure anisotropic scattering systems is related to the average cosine of the scattering angle $\left(\bar{cos\theta }\right)$ by:

(21) $$D=\frac{c}{3\left(1-\bar{cos\theta }\right)\sigma_{s}},\;\bar{cos\theta }=\int cos\theta \cdot p\left({\vec{\Omega }}^{\prime }\to \vec{\Omega }\right)d\vec{\Omega }.$$

Therefore, the diffusion coefficient of systems containing both absorption–emission and anisotropic scattering processes can be derived through a similar method:

(22) $$D=\frac{c}{3\left[\sigma_{a}+\left(1-\bar{cos\theta }\right)\sigma_{s}\right]}.$$

Hence, the macroscopic equations and macroscopic flux of the anisotropic systems remain the same structure as those in the isotropic system in (3) and (12). But the effective diffusion coefficients in macroscopic flux should be adopted:

(23) $$\left\{\begin{array}{cc} \; & \lambda_{aniso}=\frac{c\left[L_{a}^{\varepsilon }\sigma_{a}+L_{s}^{\varepsilon }\left(1-\bar{cos\theta }\right)\sigma_{s}\right]}{\varepsilon },\\ \; & D_{aniso}^{1}=-\frac{c^{3}l_{k}L_{a}^{\varepsilon }\sigma_{a}}{2\pi \Delta t\varepsilon^{3}\lambda_{aniso}^{2}}\left[\Delta t\left(1+e^{-\lambda \Delta t}\right)-\frac{2}{\lambda }\left(1-e^{-\lambda \Delta t}\right)\right],\\ \; & D_{aniso}^{2}=-\frac{c^{3}l_{k}L_{s}^{\varepsilon }\left(1-\bar{cos\theta }\right)\sigma_{s}}{2\pi \Delta t\varepsilon^{3}\lambda_{aniso}^{2}}\left[\Delta t\left(1+e^{-\lambda \Delta t}\right)-\frac{2}{\lambda }\left(1-e^{-\lambda \Delta t}\right)\right], \end{array}\right.$$

which will approach diffusion coefficient (22) within the optically thick regimes. Then, the same source iteration method in Section 2.1.2 is utilized to solve the macroscopic equations in the anisotropic-UGKP method.

#### 2.2.3. Microscopic Solver for the Anisotropic-UGKP Method

The integral solution of radiation intensity around the center of edge *k* in the anisotropic system (18) follows the same formation as in (6) expect that the scattering source is changed into *S*:

(24) $$\begin{array}{cc} I\left(t,0,0\right)= & e^{-\lambda \left(t-t_{n}\right)}I\left(t_{n},-\frac{\mu^{\prime }c}{\varepsilon }\left(t-t_{n}\right),-\frac{\xi^{\prime }c}{\varepsilon }\left(t-t_{n}\right)\right)\\ \; & +\int_{t_{n}}^{t}e^{-\lambda \left(t-s\right)}\left[\begin{array}{c} \frac{cL_{a}^{\varepsilon }\sigma_{a}}{\varepsilon }\frac{1}{2\pi }\phi \left(s,-\frac{\mu^{\prime }c}{\varepsilon }\left(t-s\right),-\frac{\xi^{\prime }c}{\varepsilon }\left(t-s\right)\right)\\ +\frac{cL_{s}^{\varepsilon }\sigma_{s}}{\varepsilon }S\left(s,-\frac{\mu^{\prime }c}{\varepsilon }\left(t-s\right),-\frac{\xi^{\prime }c}{\varepsilon }\left(t-s\right)\right) \end{array}\right]ds. \end{array}$$

Therefore, the following formation can be derived under the first-order expansion of the macroscopic quantities:

(25) $$\begin{array}{cc} \; & \begin{array}{cc} I\left(t_{n+1},x^{\prime },y^{\prime }\right) & =e^{-\lambda \Delta t}I\left(t_{n},x^{\prime }-\frac{\mu^{\prime }c}{\varepsilon }\Delta t,y^{\prime }-\frac{\xi^{\prime }c}{\varepsilon }\Delta t\right)+\frac{1}{\lambda }\left(1-e^{-\lambda \Delta t}\right)\left[\frac{cL_{a}^{\varepsilon }\sigma_{a}}{\varepsilon }\frac{\phi_{j}^{n+1}}{2\pi }+\frac{cL_{s}^{\varepsilon }\sigma_{s}}{\varepsilon }S_{j}^{n+1}\right]\\ \; & =e^{-\lambda \Delta t}I^{transport}\left(t_{n+1},x^{\prime },y^{\prime }\right)+\left(1-e^{-\lambda \Delta t}\right){\left(I^{diffusion}\right)}_{j}^{n+1}, \end{array}\\ \; & I^{transport}\left(t_{n+1},x^{\prime },y^{\prime }\right)=I\left(t_{n},x^{\prime }-\frac{\mu^{\prime }c}{\varepsilon }\Delta t,y^{\prime }-\frac{\xi^{\prime }c}{\varepsilon }\Delta t\right),\\ \; & {\left(I^{diffusion}\right)}_{j}^{n+1}=\frac{1}{\lambda }\left[\frac{cL_{a}^{\varepsilon }\sigma_{a}}{\varepsilon }\frac{\phi_{j}^{n+1}}{2\pi }+\frac{cL_{s}^{\varepsilon }\sigma_{s}}{\varepsilon }S_{j}^{n+1}\right]. \end{array}$$

Similar to (15), the re-emitted and scattered photons are re-sampled from $\left(1-e^{-\lambda \Delta t}\right)$ ${\left(I^{diffusion}\right)}_{j}^{n+1}$ only. The re-emitted photons are re-sampled in a similar manner in the isotropic-UGKP method. The scattered photons, however, need to be sampled with the same angular distribution as $S_{j}^{n+1}$ .

Based on Equation (20), $S_{j}^{n+1}$ can be written as:

(26) $$S_{j}^{n+1}=\sum_{l=0}^{L}C_{l}^{*}\sum_{i_{1}i_{2}\cdots i_{l}}\left[\Omega_{i_{1}i_{2}\cdots i_{l}}^{\left(l\right)}{\left(J_{i_{1}i_{2}\cdots i_{l}}^{\left(l\right)}\right)}_{j}^{n+1}\right],$$

which is related to moments ${\left(J^{\left(l\right)}\right)}_{j}^{n+1}$ with 0≤l≤L . Thus, one common way is constructing the UGKP method for all these moments above. In doing so, the macroscopic system must contain the macroscopic equation of all moments’ components, and the corresponding macroscopic flux of each macroscopic equation need also be calculated. As a result, the calculating costs will increase exponentially with the highest order *L* increasing (since the total components’ number of $J^{\left(l\right)}$ is $3^{l}$ ). What is more, $S_{j}^{n+1}$ has a complex relationship with moments, making it challenging to directly sample the angular distribution from $S_{j}^{n+1}$ .

To overcome these issues, we propose a revised phase function as an equivalent to the calculation and angular sampling of $S_{j}^{n+1}$ , drawing on our prior theoretical work [41]. This equivalent phase function, which will be derivated and discussed in Section 2.2.4 in detail, incorporates a factor *f* applied to all odd-order coefficients of the original phase function (19):

(27) $$\left\{\begin{array}{cc} \; & p_{eq}\left({\vec{\Omega }}^{\prime }\to \vec{\Omega }\right)=\sum_{l=even}^{L}C_{l}^{*}{\left(\vec{\Omega }\cdot {\vec{\Omega }}^{\prime }\right)}^{l}+f\sum_{l=odd}^{L}C_{l}^{*}{\left(\vec{\Omega }\cdot {\vec{\Omega }}^{\prime }\right)}^{l},\\ \; & f=\frac{e^{-\lambda \Delta t}}{1-\frac{1}{\lambda }\left(1-e^{-\lambda \Delta t}\right)\frac{cL_{s}^{\varepsilon }\sigma_{s}}{\varepsilon }\bar{cos\theta }}. \end{array}\right.$$

The factor *f* varies between 0 and 1 in the diffusion and free transport limit regimes, respectively.

With (27), we introduce modifications to the photon tracking and re-sampling processes within the microscopic solver of the anisotropic-UGKP method. In the anisotropic-UGKP method, photons are freely transported in the time step $t\in \left[t_{n},t_{n+1}\right]$ first. At the end of time step $t_{n+1}$ , photons are then sampled to be freely transported (with probability $e^{-\lambda \Delta t}$ in Equation (25)), absorbed (with probability $\left(1-e^{-\lambda \Delta t}\right)\frac{1}{\lambda }\frac{cL_{a}^{\varepsilon }\sigma_{a}}{\varepsilon }$ in Equation (25)), or scattered (with probability $\left(1-e^{-\lambda \Delta t}\right)\frac{1}{\lambda }\frac{cL_{s}^{\varepsilon }\sigma_{s}}{\varepsilon }$ in Equation (25)), which is based on the current mesh’s cross-sections. The freely streamed and re-emitted photons are treated in a similar way as in the isotropic-UGKP method. The scattered photons, in contrast, will be redirected into a new direction, with the scattering angle sampled from (27). Since photons are freely transported during the photon tracking, the $\Phi_{j,k}^{n+1,micro}$ term of macroscopic flux is also adapted:

(28) $$\begin{array}{cc} \; & \Phi_{j,k}^{n+1,micro}=e^{-\lambda_{k}\Delta t}\sum_{i}1\left(\mu^{\prime }\right)w_{i},\;\lambda_{k}=\frac{c\left[L_{a}^{\varepsilon }{\left(\sigma_{a}\right)}_{k}+L_{s}^{\varepsilon }{\left(\sigma_{s}\right)}_{k}\right]}{\varepsilon },\\ \; & {\left(\sigma_{a}\right)}_{k}=\frac{2{\left(\sigma_{a}\right)}_{j}{\left(\sigma_{a}\right)}_{j^{\prime }}}{{\left(\sigma_{a}\right)}_{j}+{\left(\sigma_{a}\right)}_{j^{\prime }}},\;{\left(\sigma_{s}\right)}_{k}=\frac{2{\left(\sigma_{s}\right)}_{j}{\left(\sigma_{s}\right)}_{j^{\prime }}}{{\left(\sigma_{s}\right)}_{j}+{\left(\sigma_{s}\right)}_{j^{\prime }}}. \end{array}$$

With this adapted microscopic solver, there is no need to calculate the macroscopic fluxes and solve the macroscopic equations of high-order moments, significantly reducing the computational costs. The scattered photons are also easily re-sampled with (27) compared with directly sampling the angular distribution from $S_{j}^{n+1}$ . This adapted microscopic solver is summarized by the following Algorithm 3:
**Algorithm 3** Photon re-sampling and tracking algorithm for the adapted microscopic radiation intensity evolution.
1:Photon re-sampling:2:**for all** photon **do**3:   Sample the photon’s status: freely transported, absorbed, or scattered.4:   **if** Photon is freely transported **then**5:     Continually simulate the photon in the next time step.6:   **else if** Photon is absorbed **then**7:     Remove the photon from the simulation.8:   **else if** Photon is scattered **then**9:     Sample the scattering angle from (27) and redirect the photon.10:  **end if**11:**end for**12:**for all** mesh cell **do**13:  Re-sample the re-emitted photons isotropically and uniformly in mesh cell.14:**end for**15: 16:Photon tracking:17:**for all** photon **do**18:  **repeat**19:    Track photon and calculate the $\Phi_{j,k}^{n+1,micro}$ by (28).20:  **until** Photon leaks out of the system **or** Photon reaches the end of time step21:  **if** Photon leaks out of the system **then**22:    Remove the photon from the simulation.23:   **end if**24:**end for**


#### 2.2.4. Derivation and Discussion of the Equivalent Multiscale Phase Function

In this subsection, we commence with the derivation of the equivalent multiscale phase function (27). Then, we address the normalization condition, non-negativity condition, and multiscale property of (27).

**Proposition** **1.** *The discretized scattering source $S_{j}^{n+1}$ satisfies:*

(29) $$\begin{array}{cc} \; & S_{j}^{n+1}=\frac{1}{V_{j}}\int_{V_{j}}\int p_{eq}\left({\vec{\Omega }}^{\prime }\to \vec{\Omega }\right)I^{transport}\left(t_{n+1},\vec{r},{\vec{\Omega }}^{\prime }\right)d{\vec{\Omega }}^{\prime }dxdy, \end{array}$$

*with $p_{eq}\left({\vec{\Omega }}^{\prime }\to \vec{\Omega }\right)$ as the equivalent phase function *(27)*. The $I^{transport}$ here stands for the freely transported radiation intensity during a time step $\left[t_{n},t_{n+1}\right]$ .*

**Proof** **of** **Proposition** **1.** Based on the definition of *S* and Equation (20), the discretized scattering source $S_{j}^{n+1}$ equals:

(30) $$S_{j}^{n+1}=\frac{1}{V_{j}}\int_{V_{j}}\int p\left({\vec{\Omega }}^{\prime }\to \vec{\Omega }\right)I\left(t_{n+1},\vec{r},{\vec{\Omega }}^{\prime }\right)d{\vec{\Omega }}^{\prime }dxdy=\sum_{l=0}^{L}C_{l}^{*}\sum_{i_{1}i_{2}\cdots i_{l}}\left(\Omega_{i_{1}i_{2}\cdots i_{l}}^{\left(l\right)}{\left(J_{i_{1}i_{2}\cdots i_{l}}^{\left(l\right)}\right)}_{j}^{n+1}\right).$$

With Equation (25), the components of moments $J^{\left(l\right)}$ have the form:

(31) $$\left\{\begin{array}{cc} \; & \begin{array}{cc} {\left(J_{i_{1}\cdots i_{l}}^{\left(l=even\right)}\right)}_{j}^{n+1}\; & =e^{-\lambda \Delta t}\underset{\mathrm{free}\;\mathrm{transport}}{\underset{︸}{\frac{1}{V_{j}}\int \langle \Omega_{i_{1}\cdots i_{l}}^{\left(l=even\right)}I\left(t_{n},\vec{r}-\frac{\vec{\Omega }c}{\varepsilon }\Delta t,\vec{\Omega }\right)\rangle dxdy}}\\ \; & +\left(1-e^{-\lambda \Delta t}\right)\underset{\mathrm{diffusion}}{\underset{︸}{\frac{1}{\lambda }\left[\frac{cL_{a}^{\varepsilon }\sigma_{a}}{\varepsilon }\frac{\langle \Omega_{i_{1}\cdots i_{l}}^{\left(l=even\right)}\rangle }{\langle \Omega^{0}\rangle }\phi_{j}^{n+1}+\frac{cL_{s}^{\varepsilon }\sigma_{s}}{\varepsilon }{\left(S_{i_{1}\cdots i_{l}}^{\left(l=even\right)}\right)}_{j}^{n+1}\right]}}, \end{array}\\ \; & \begin{array}{cc} {\left(J_{i_{1}\cdots i_{l}}^{\left(l=odd\right)}\right)}_{j}^{n+1}\; & =e^{-\lambda \Delta t}\underset{\mathrm{free}\;\mathrm{transport}}{\underset{︸}{\frac{1}{V_{j}}\int \langle \Omega_{i_{1}\cdots i_{l}}^{\left(l=odd\right)}I\left(t_{n},\vec{r}-\frac{\vec{\Omega }c}{\varepsilon }\Delta t,\vec{\Omega }\right)\rangle dxdy}}\\ \; & +\left(1-e^{-\lambda \Delta t}\right)\underset{\mathrm{diffusion}}{\underset{︸}{\frac{1}{\lambda }\frac{cL_{s}^{\varepsilon }\sigma_{s}}{\varepsilon }{\left(S_{i_{1}\cdots i_{l}}^{\left(l=odd\right)}\right)}_{j}^{n+1}}}, \end{array} \end{array}\right.$$

where $S^{\left(l\right)}$ is the *l*th-order moments of *S*, and $S_{i_{1}i_{2}\cdots i_{l}}^{\left(l\right)}$ is the $\left\{i_{1}i_{2}\cdots i_{l}\right\}$ component of $S^{\left(l\right)}$ . The even-order moments have the following relationship for all flow regimes [41]:

(32) $$S_{i_{1}\cdots i_{l}}^{\left(l=even\right)}=J_{i_{1}\cdots i_{l}}^{\left(l=even\right)}=\frac{\langle \Omega_{i_{1}\cdots i_{l}}^{\left(l=even\right)}\rangle }{\langle \Omega^{\left(0\right)}\rangle }\rho ,\;0\leq l\leq L.$$

In the diffusion limit, we have [37,41]:

(33) $$\phi_{j}^{n+1}=\rho_{j}^{n+1},\;{\left(S_{i_{1}\cdots i_{l}}^{\left(l=odd\right)}\right)}_{j}^{n+1}=\bar{cos\theta }{\left(J_{i_{1}\cdots i_{l}}^{\left(l=odd\right)}\right)}_{j}^{n+1}.$$

Thus, the components of moments $J^{\left(l\right)}$ can be written as:

(34) $$\left\{\begin{array}{cc} \; & {\left(J_{i_{1}\cdots i_{l}}^{\left(l=even\right)}\right)}_{j}^{n+1}=\frac{1}{V_{j}}\int \langle \Omega_{i_{1}\cdots i_{l}}^{\left(l=even\right)}I\left(t_{n},\vec{r}-\frac{\vec{\Omega }c}{\varepsilon }\Delta t,\vec{\Omega }\right)\rangle dxdy={\left(J_{i_{1}i_{2}\cdots i_{l}}^{\left(l=even\right)}\right)}_{j}^{n+1,transport},\\ \; & {\left(J_{i_{1}\cdots i_{l}}^{\left(l=odd\right)}\right)}_{j}^{n+1}=f\left[\frac{1}{V_{j}}\int \langle \Omega_{i_{1}\cdots i_{l}}^{\left(l=odd\right)}I\left(t_{n},\vec{r}-\frac{\vec{\Omega }c}{\varepsilon }\left(t-t_{n}\right),\vec{\Omega }\right)\rangle dxdy\right]=f{\left(J_{i_{1}\cdots i_{l}}^{\left(l=odd\right)}\right)}_{j}^{n+1,transport}. \end{array}\right.$$

Therefore, Equation (29) can be proved:

(35) $$\begin{array}{cc} S_{j}^{n+1} & =\sum_{l=even}^{L}C_{l}^{*}\sum_{i_{1}i_{2}\cdots i_{l}}^{l=even}\left(\Omega_{i_{1}i_{2}\cdots i_{l}}^{\left(l=even\right)}{\left(J_{i_{1}i_{2}\cdots i_{l}}^{\left(l=even\right)}\right)}_{j}^{n+1}\right)+\sum_{l=odd}^{L}C_{l}^{*}\sum_{i_{1}i_{2}\cdots i_{l}}^{l=odd}\left(\Omega_{i_{1}i_{2}\cdots i_{l}}^{\left(l=odd\right)}{\left(J_{i_{1}i_{2}\cdots i_{l}}^{\left(l=odd\right)}\right)}_{j}^{n+1}\right)\\ \; & =\sum_{l=even}^{L}C_{l}^{*}\sum_{i_{1}i_{2}\cdots i_{l}}^{l=even}\left(\Omega_{i_{1}i_{2}\cdots i_{l}}^{\left(l=even\right)}{\left(J_{i_{1}i_{2}\cdots i_{l}}^{\left(l=even\right)}\right)}_{j}^{n+1,transport}\right)+f\sum_{l=odd}^{L}C_{l}^{*}\sum_{i_{1}i_{2}\cdots i_{l}}^{l=odd}\left(\Omega_{i_{1}i_{2}\cdots i_{l}}^{\left(l=odd\right)}{\left(J_{i_{1}i_{2}\cdots i_{l}}^{\left(l=odd\right)}\right)}_{j}^{n+1,transport}\right)\\ \; & =\frac{1}{V_{j}}\int_{V_{j}}\int p_{eq}\left({\vec{\Omega }}^{\prime }\to \vec{\Omega }\right)I^{transport}\left(t_{n+1},\vec{r},{\vec{\Omega }}^{\prime }\right)d{\vec{\Omega }}^{\prime }dxdy, \end{array}$$

where a reverse derivation of Equation (20) is used. Based on Equation (35), the angular distribution of scattering source $S_{j}^{n+1}$ can be equivalent to one single scattering process with (27), saving the computational costs.    □

**Proposition** **2.** *The equivalent multiscale phase function *(27)* satisfies the normalization condition:*

(36) $$\int p_{eq}\left({\vec{\Omega }}^{\prime }\to \vec{\Omega }\right)d{\vec{\Omega }}^{\prime }=1.$$

**Proof** **of** **Proposition** **2.** For the original phase function (19), it satisfies the normalization condition:

(37) $$\int p\left({\vec{\Omega }}^{\prime }\to \vec{\Omega }\right)d{\vec{\Omega }}^{\prime }=\sum_{l=0}^{L}C_{l}^{*}\langle {\left(cos\theta \right)}^{l}\rangle =\sum_{l=even}^{L}C_{l}^{*}\langle {\left(cos\theta \right)}^{l}\rangle =1.$$

What is more, the normalization condition of the phase function is only dependent on the even-order coefficients $C_{l=even}^{*}$ . Hence, with identical even-order coefficients, the equivalent multiscale phase function satisfies the normalization condition as the original one.    □

**Proposition** **3.** *The equivalent multiscale phase function *(27)* satisfies the non-negativity condition:*

(38) $$p_{eq}\left({\vec{\Omega }}^{\prime }\to \vec{\Omega }\right)=\sum_{l=even}^{L}C_{l}^{*}{\left(cos\theta \right)}^{l}+f\sum_{l=odd}^{L}C_{l}^{*}{\left(cos\theta \right)}^{l}\geq 0,\;for\;any\;\;\theta .$$

**Proof** **of** **Proposition** **3.** To substantiate the non-negativity condition (38), the range of factor *f* needs to be determined first. Taking $L_{a}^{\varepsilon }=L_{s}^{\varepsilon }$ , the factor *f* has the following form:

(39) $$f=\frac{e^{-\lambda \Delta t}}{1-\frac{1}{\lambda }\left(1-e^{-\lambda \Delta t}\right)\frac{cL_{s}^{\varepsilon }\sigma_{s}}{\varepsilon }\bar{cos\theta }}=\frac{e^{-\lambda \Delta t}}{1-\left(1-e^{-\lambda \Delta t}\right)\omega \bar{cos\theta }},$$

where $\omega =\sigma_{s}/\left(\sigma_{a}+\sigma_{s}\right)$ is the scattering albedo with 0≤ω≤1 , and $-1<\bar{cos\theta }<1$ . For all cases below with ω≠0 , the factor *f* satisfies 0≤f≤1 :

(40) $$\left\{\begin{array}{cc} \; & e^{-\lambda \Delta t}=1\Rightarrow f=1,\\ \; & 0<e^{-\lambda \Delta t}<1:\left\{\begin{array}{cc} \; & \begin{array}{cc} 0<\bar{cos\theta }<1:\; & \left(1-e^{-\lambda \Delta t}\right)\bar{cos\theta }<\left(1-e^{-\lambda \Delta t}\right)\to \frac{e^{-\lambda \Delta t}}{1-\left(1-e^{-\lambda \Delta t}\right)\bar{cos\theta }}<1\\ \; & 1-\left(1-e^{-\lambda \Delta t}\right)\omega \bar{cos\theta }\geq 1-\left(1-e^{-\lambda \Delta t}\right)\bar{cos\theta }>0\\ \; & \Rightarrow 0<f=\frac{e^{-\lambda \Delta t}}{1-\left(1-e^{-\lambda \Delta t}\right)\omega \bar{cos\theta }}<\frac{e^{-\lambda \Delta t}}{1-\left(1-e^{-\lambda \Delta t}\right)\bar{cos\theta }}<1, \end{array}\\ \; & \begin{array}{cc} -1<\bar{cos\theta }<0:\; & 1-\left(1-e^{-\lambda \Delta t}\right)\omega \bar{cos\theta }>1\to \frac{1}{1-\left(1-e^{-\lambda \Delta t}\right)\omega \bar{cos\theta }}<1\\ \; & \Rightarrow 0<f=\frac{e^{-\lambda \Delta t}}{1-\left(1-e^{-\lambda \Delta t}\right)\omega \bar{cos\theta }}<1, \end{array} \end{array}\right.\\ \; & e^{-\lambda \Delta t}=0\Rightarrow f=0. \end{array}\right.$$

For the special case with ω=0 , the scattering process does not exist, while another special case with $\bar{cos\theta }=0$ stands for a phase function without any odd-order terms. Hence, the odd-order terms of (27) are not considered in either of these two cases.

For the original phase function (19), it satisfies the following formation:

(41) $$p\left({\vec{\Omega }}^{\prime }\to \vec{\Omega }\right)=\sum_{l=even}^{L}C_{l}^{*}{\left(cos\theta \right)}^{l}+\sum_{l=odd}^{L}C_{l}^{*}{\left(cos\theta \right)}^{l}\geq 0,\;\mathrm{for}\;\mathrm{any}\;\theta ,$$

which gives:

(42) $$\left\{\begin{array}{cc} \; & \sum_{l=even}^{L}C_{l}^{*}{\left(cos\alpha \right)}^{l}+\sum_{l=odd}^{L}C_{l}^{*}{\left(cos\alpha \right)}^{l}\geq 0,\;\theta =\alpha \in \left[0,\frac{\pi }{2}\right],\\ \; & \sum_{l=even}^{L}C_{l}^{*}{\left(cos\alpha \right)}^{l}-\sum_{l=odd}^{L}C_{l}^{*}{\left(cos\alpha \right)}^{l}\geq 0,\;\theta =\pi -\alpha , \end{array}\right.\Rightarrow \sum_{l=even}^{L}C_{l}^{*}{\left(cos\theta \right)}^{l}\geq 0,\;\mathrm{for}\;\mathrm{any}\;\theta$$

Therefore, the non-negativity condition (38) can be proved based on Equations (40), (41) and (42):

(43) $$\left\{\begin{array}{cc} \; & \sum_{l=odd}^{L}C_{l}^{*}{\left(cos\theta \right)}^{l}\geq 0:\sum_{l=even}^{L}C_{l}^{*}{\left(cos\theta \right)}^{l}+f\sum_{l=odd}^{L}C_{l}^{*}{\left(cos\theta \right)}^{l}\geq 0,\\ \; & \sum_{l=odd}^{L}C_{l}^{*}{\left(cos\theta \right)}^{l}<0:\sum_{l=even}^{L}C_{l}^{*}{\left(cos\theta \right)}^{l}-f\left|\sum_{l=odd}^{L}C_{l}^{*}{\left(cos\theta \right)}^{l}\right|\geq \sum_{l=even}^{L}C_{l}^{*}{\left(cos\theta \right)}^{l}-\left|\sum_{l=odd}^{L}C_{l}^{*}{\left(cos\theta \right)}^{l}\right|\geq 0. \end{array}\right.$$

   □

**Proposition** **4.** *The equivalent multiscale phase function *(27)* has a multiscale property:*

*1*  *In the optically thin regime, the equivalent multiscale phase function *(27)* degenerates to the original phase function *(19)*;*
*2*  *In the optically thick regime, the equivalent multiscale phase function *(27)* preserves the isotropy of the scattering source.*

**Proof** **of** **Proposition** **4.** As indicated in Equation (40), the factor *f* ranges from 0 to 1 in the diffusion and free transport limit regimes, rendering the equivalent phase function (27) a multiscale phase function. The factor *f* equals 1 in the optically thin regime as λ→0 . Thus, the equivalent multiscale phase function (27) aligns with the original phase function (19). With λ increasing, more frequent collision processes lead to a reduction in the anisotropy of the macroscopic scattering source, which is signified by the factor *f* decreasing. In the optically thick regime as λ→∞ , the factor *f* tends toward 0, causing all odd-order coefficients in (27) to approach 0. Therefore, the equivalent multiscale phase function (27) transforms into a phase function without any odd-order terms, preserving the isotropy of the macroscopic scattering source [41].    □

#### 2.2.5. Summary for the Anisotropic-UGKP Method

For the anisotropic-UGKP method, the microscopic solver furnishes the free-streaming numerical flux for solving the macroscopic equations, and the solution provided by the macroscopic solver serves as the closure source for the microscopic intensity. The macroscopic and microscopic solver are closely coupled for solving the macroscopic energy and microscopic intensity. The anisotropic-UGKP method is concisely summarized as following Algorithm 4.
**Algorithm 4** The algorithm for the anisotropic UGKP method.
1:Initialize the flow field and sample photon particles.2:**for** Simulation time less than the final time **do**3:   Stream all particles by the microscopic solver, and calculate the free-streaming flux (28).4:   Apply the macroscopic solver to evolve the macroscopic quantities.5:   Re-sample the re-emitted and scattered source photons and recover the microscopic radiation intensity with Equation (25).6:**end for**


### 2.3. Asymptotic Analysis

The asymptotic preserving (AP) property is important for the construction of a multiscale scheme. For the free transport and single-temperature diffusive regimes, the asymptotic preserving analysis is similar to that in our previous work [37]. For brevity, we omit the repetition of those details here. In this subsection, we focus on demonstrating the asymptotic preserving property of the proposed anisotropic-UGKP method in the other diffusion regimes.

**Proposition** **5.** *The proposed anisotropic-UGKP method preserves the two-temperature diffusion system:*

(44) $$\left\{\begin{array}{cc} \; & \frac{\partial \rho }{\partial t}-\nabla \cdot \frac{c}{3\left(1-\bar{cos\theta }\right)\sigma_{s}}\nabla \rho =c\sigma_{a}\left(\phi -\rho \right),\\ \; & C_{V}\frac{\partial T}{\partial t}\equiv \frac{\partial U_{m}}{\partial t}=\sigma_{a}\left(\rho -\phi \right). \end{array}\right.$$

**Proof** **of** **Proposition** **5.** For the two-temperature diffusion regime or non-equilibrium diffusion limit, the Knudsen number ε approaches 0, with $L_{a}^{\varepsilon }=\varepsilon$ and Lsε=11εε . The effective diffusion coefficients in (23) have the following orders:

(45) λ=cεσa+σsσsεεε→oε−2,λaniso=cεσa+1−cosθ¯σs/εε,e−λΔt→0,Daniso1j,kn+1→−cσaj,kn+1lk2πεσaj,kn+1+1−cosθ¯σsj,kn+1σsj,kn+1εε2→oε2→0,Daniso2j,kn+1→−c1−cosθ¯σsj,kn+1lk2πε2εσaj,kn+1+1−cosθ¯σsj,kn+1σsj,kn+1εε2→−clk2π1−cosθ¯σsj,kn+1.

Then, the macroscopic numerical flux (12) for an anisotropic system has the following form:

(46) $$\Phi_{j,k}^{n+1}\to -\frac{cl_{k}}{3\left(1-\bar{cos\theta }\right){\left(\sigma_{s}\right)}_{j,k}^{n+1}}\delta_{x^{\prime }}\rho_{j,k}^{n+1}.$$

Substituting the macroscopic numerical flux (46) into the macroscopic Equation (3), we have

(47) $$\begin{array}{c} \rho_{j}^{n+1}=\rho_{j}^{n}-\frac{\Delta t}{V_{j}}\sum_{k}\left(-\frac{cl_{k}}{3\left(1-\bar{cos\theta }\right){\left(\sigma_{s}\right)}_{j,k}^{n+1}}\delta_{x^{\prime }}\rho_{j,k}^{n+1}\right)+c\Delta t{\left(\sigma_{a}\right)}_{j}^{n+1}\left(\phi_{j}^{n+1}-\rho_{j}^{n+1}\right) \end{array}$$

Equation (47) is a standard nine-point scheme for the diffusion limit equation in (44) for an anisotropic system independent of the parameter ε . The second equation of (44) is also independent of the parameter ε under $L_{a}^{\varepsilon }=\varepsilon$ and Lsε=11εε . Thus, the convergence of Equation (44) is inherently satisfied. Furthermore, the equivalent multiscale phase function (27) also maintains the isotropy of the angular distribution of macroscopic scattering source in the diffusion regime. The above discussions affirm that the proposed anisotropic-UGKP method preserves the two-temperature non-equilibrium diffusion limit. □

**Proposition** **6.** *For systems with absorption–emission and scattering processes equally dominated, the proposed anisotropic-UGKP method preserves the following diffusion equation:*

(48) $$\frac{\partial }{\partial t}U_{m}\left(T\right)+\frac{\partial }{\partial t}\left(aT^{4}\right)=\nabla \cdot \frac{1}{3\left[\sigma_{a}+\left(1-\bar{cos\theta }\right)\sigma_{s}\right]}\nabla \left(acT^{4}\right).$$

**Proof** **of** **Proposition** **6.** For systems with absorption–emission and scattering processes equally dominated, the Knudsen number ε approaches 0 and Laε=Lsε∼11εε . The effective diffusion coefficients in (23) have the following orders:

(49) $$\begin{array}{cc} \; & \lambda =\frac{c\left(\sigma_{a}+\sigma_{s}\right)}{\varepsilon^{2}}∼O\left(\varepsilon^{-2}\right),\;\;\lambda_{aniso}=\frac{c\left[\sigma_{a}+\left(1-\bar{cos\theta }\right)\sigma_{s}\right]}{\varepsilon^{2}},\;\;e^{-\lambda \Delta t}\to 0,\\ \; & {\left(D_{aniso}^{1}\right)}_{j,k}^{n+1}\to -\frac{c{\left(\sigma_{a}\right)}_{j,k}^{n+1}l_{k}}{2\pi {\left[{\left(\sigma_{a}\right)}_{j,k}^{n+1}+\left(1-\bar{cos\theta }\right){\left(\sigma_{s}\right)}_{j,k}^{n+1}\right]}^{2}},\\ \; & {\left(D_{aniso}^{2}\right)}_{j,k}^{n+1}\to -\frac{c\left(1-\bar{cos\theta }\right){\left(\sigma_{s}\right)}_{j,k}^{n+1}l_{k}}{2\pi {\left[{\left(\sigma_{a}\right)}_{j,k}^{n+1}+\left(1-\bar{cos\theta }\right){\left(\sigma_{s}\right)}_{j,k}^{n+1}\right]}^{2}}. \end{array}$$

Therefore, the macroscopic numerical flux (49) has the following form:

(50) $$\Phi_{j,k}^{n+1}\to -\frac{c{\left(\sigma_{a}\right)}_{j,k}^{n+1}l_{k}}{3{\left[{\left(\sigma_{a}\right)}_{j,k}^{n+1}+\left(1-\bar{cos\theta }\right){\left(\sigma_{s}\right)}_{j,k}^{n+1}\right]}^{2}}\delta_{x^{\prime }}\phi_{j,k}^{n+1}-\frac{c\left(1-\bar{cos\theta }\right){\left(\sigma_{s}\right)}_{j,k}^{n+1}l_{k}}{3{\left[{\left(\sigma_{a}\right)}_{j,k}^{n+1}+\left(1-\bar{cos\theta }\right){\left(\sigma_{s}\right)}_{j,k}^{n+1}\right]}^{2}}\delta_{x^{\prime }}\rho_{j,k}^{n+1}.$$

Apply the asymptotic analysis to the macroscopic Equation (3), and we have the $O\left(\varepsilon^{-2}\right)$ equation:

(51) $$\phi_{j}^{n+1}=\rho_{j}^{n+1}.$$

Therefore, the macroscopic numerical flux (50) can be rewritten as:

(52) $$\Phi_{j,k}^{n+1}\to -\frac{cl_{k}}{3\left[{\left(\sigma_{a}\right)}_{j,k}^{n+1}+\left(1-\bar{cos\theta }\right){\left(\sigma_{s}\right)}_{j,k}^{n+1}\right]}\delta_{x^{\prime }}\phi_{j,k}^{n+1}.$$

Coupling the macroscopic numerical flux (50) and the macroscopic Equation (3), we derive the $O\left(\varepsilon^{0}\right)$ equation:

(53) $$\begin{array}{c} \rho_{j}^{n+1}=\rho_{j}^{n}-\frac{\Delta t}{V_{j}}\sum_{k}\left(-\frac{cl_{k}}{3\left[{\left(\sigma_{a}\right)}_{j,k}^{n+1}+\left(1-\bar{cos\theta }\right){\left(\sigma_{s}\right)}_{j,k}^{n+1}\right]}\delta_{x^{\prime }}\phi_{j,k}^{n+1}\right)-cC_{V}\left(T_{j}^{n+1}-T_{j}^{n}\right). \end{array}$$

It shows that Equation (53) becomes a standard nine-point scheme for the diffusion limit Equation (48) coupled with (51). The equivalent multiscale phase function (27) keeps the isotropy of the angular distribution of the macroscopic scattering source as well. Hence, the above discussions confirm that the proposed anisotropic-UGKP method preserves the diffusion limit equation for systems with absorption–emission and scattering processes equally dominated. □

## 3. Numerical Tests

Although our anisotropic-UGKP method is proposed for thermal radiative transfer problems, it is also suitable for neutron transport problems with pure scattering (since they have the same formation). Therefore, we present three numerical examples to validate the proposed anisotropic-UGKP method in this section, including a 1D neutron problem and two 2D radiation heat transfer problems. In the following examples, the unit of the length is taken to be centimeter (cm), the mass unit is gram (g), the time unit is nanosecond (ns), the temperature unit is kilo electronvolt (keV), and the energy unit is $10^{9}$ Joules (GJ). Under the above units, the speed of light is 29.98 cm/ns, and the radiation constant *a* is 0.01372 GJ/( ${\mathrm{cm}}^{3}$ ${\mathrm{keV}}^{4}$ ).

### 3.1. One-Dimensional (1D) Neutron Problem

In this 1D neutron problem, we consider the following initial condition and two different cross-sections:

(54) $$\begin{array}{cc} \; & I\left(x,t=0,\mu \right)=\left\{\begin{array}{cc} \; & 2,0.8<x<1.2,\\ \; & 0,otherwise, \end{array}\right.\\ \; & caseA:\sigma_{s}\left(x\right)=\left\{\begin{array}{cc} \; & 0.02\sigma ,x\in \left[0.35,0.65\right]\cup \left[1.35,1.65\right],\\ \; & \sigma ,x\in \left[0,0.35\right)\cup \left(0.65,1.35\right)\cup \left(1.65,2\right], \end{array}\right.\\ \; & caseB:\sigma_{s}\left(x\right)=100{\left(x-1\right)}^{4}\sigma , \end{array}$$

with the computational domain as $x\in \left[0,2\right]$ . This neutron problem is a pure scattering problem with $\omega =\sigma_{s}/\left(\sigma_{a}+\sigma_{s}\right)=1$ . To verify the AP property of the anisotropic-UGKP method, we investigate two scenarios: one with σ=1 and the other with $\sigma =10^{3}$ in (54). These cases represent the free transport and diffusion limit, respectively. The neutron speed is taken as 1, and the simulation time is 1 and 100 for the transport and diffusion limit. For case B with $\sigma =10^{3}$ , the cross-section spans a vast range from 0 to $10^{5}$ , covering a large range from the free transport regime to the diffusive regime. Thus, the computing is very challenging.

**Figure 2:**
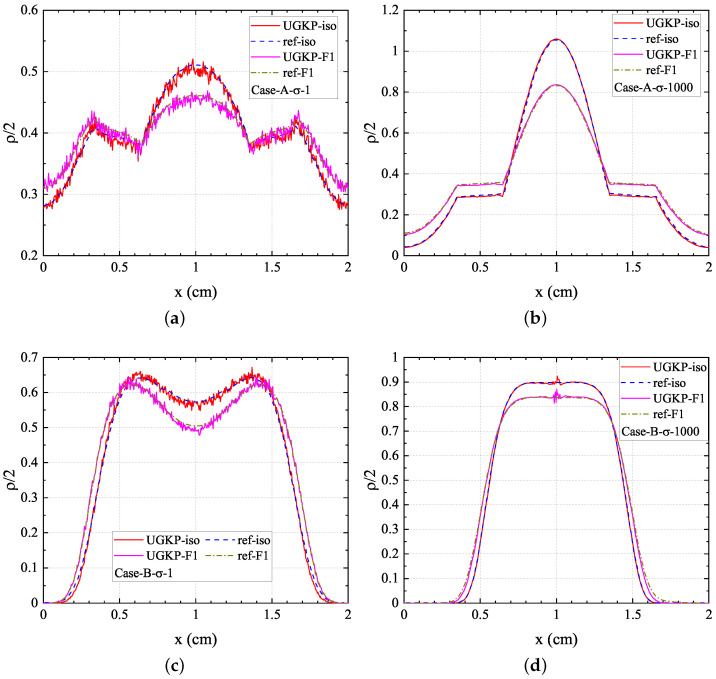
The results of 1D neutron problem with isotropic and anisotropic scattering. Case A with (**a**) σ=1 , (**b**) $\sigma =10^{3}$ ; Case B with (**c**) σ=1 , (**d**) $\sigma =10^{3}$ .

The isotropic scattering case is simulated first to verify the codes, whose results are given in Figure 2 and compared with the reference results in [42]. Subsequently, the anisotropic scattering case with the following F1 phase function is conducted to verify the anisotropic-UGKP method:

(55) $$p\left({\vec{\Omega }}^{\prime }\to \vec{\Omega }\right)=C\left(1+{\vec{\Omega }}^{\prime }\cdot \vec{\Omega }\right),$$

with *C* as the normalization factor. Figure 2 also displays a comparison between the simulation results and reference results in [42] for this F1 scattering case. As can be seen, good agreement is observed for isotropic and F1 scattering cases in both kinetic and diffusion regimes. The cross-section at x=1 is perpetually 0 for case B, switching the anisotropic-UGKP method to full MC simulation around x=1 , and therefore leading to the statistical fluctuations around x=1 .

### 3.2. Two-Dimensional (2D) Radiation Heat Transfer Problem

**Figure 3:**
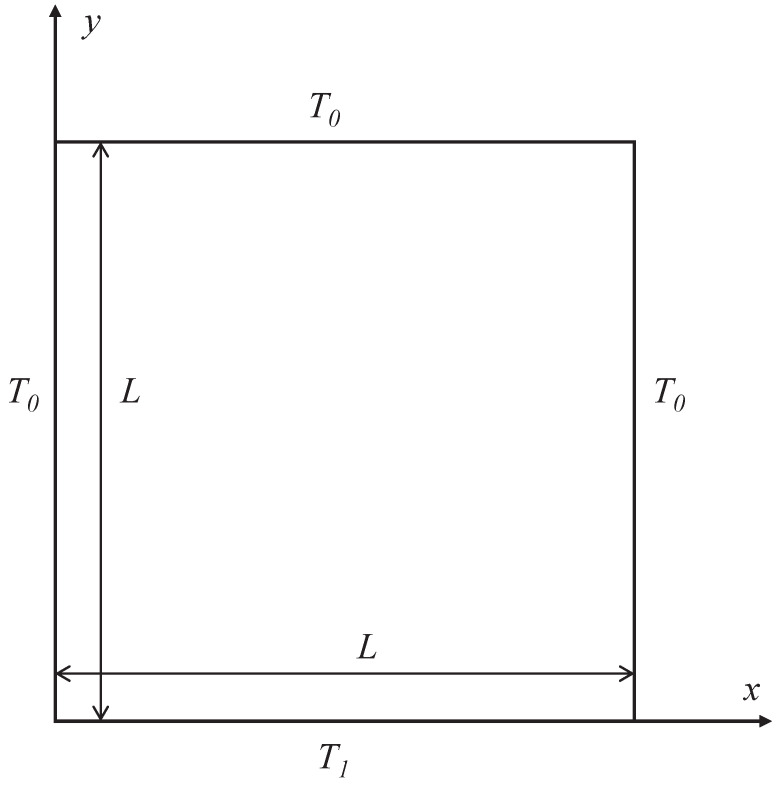
Schematic of 2D radiation heat transfer problem with a hot wall.

**Figure 4:**
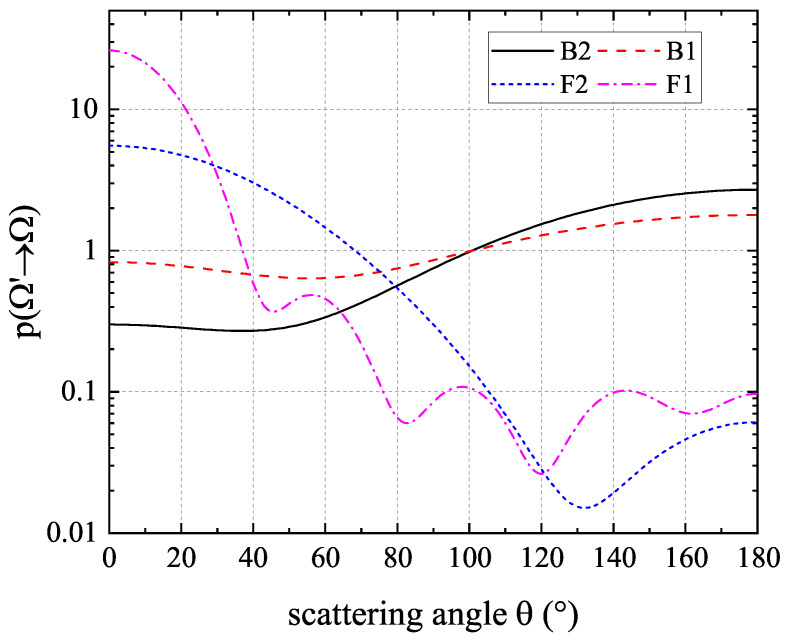
The four anisotropic phase functions varies with scattering angle θ changing in the 2D radiation heat transfer problem.

In this 2D radiation heat transfer problem, a square with the side length of 1 enclosed by four boundaries is considered, as illustrated in Figure 3. The bottom wall is kept hot with a non-dimensional temperature of $T_{1}=1$ , while all other walls are kept cold with $T_{0}=0$ . The medium is also kept cold with $T_{0}=0$ initially. Given the focus of this work on the anisotropic-UGKP method, most of the cases considered here are pure scattering (with scattering albedo ω=1 ), where the scattering process is the most significant. The isotropic scattering case is also simulated here to verify the codes, while four anisotropic cases with different phase functions are explored to verify the proposed anisotropic-UGKP method. The four anisotropic phase function varying with scattering angle θ changing are illustrated in Figure 4, and their Legendre expansion coefficients are listed in Table 1. As can be seen, the anisotropic phase functions vary significantly with the scattering angle θ changing, which leads to different transfer behavior in this problem.

**Figure 5:**
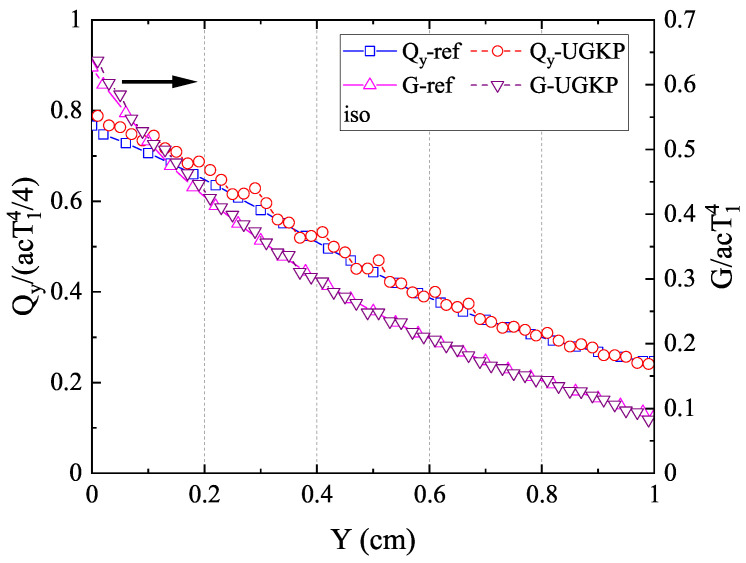
The normalized net heat flux $Q_{y}/\left(acT_{1}^{4}/4\right)$ in the *y*-direction and radiation intensity $G/acT_{1}^{4}$ along the centerline (x=0.5) for isotropic scattering compared with the reference results.

**Figure 6:**
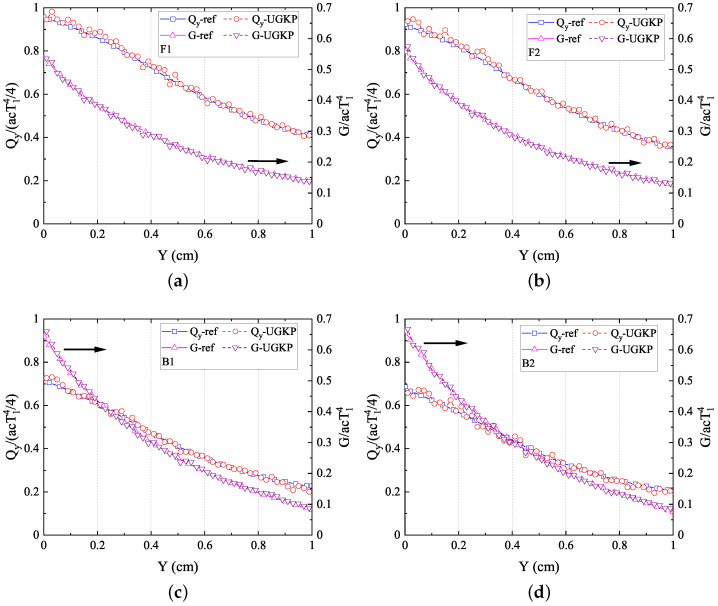
The normalized net heat flux $Q_{y}/\left(acT_{1}^{4}/4\right)$ in the *y*-direction and radiation intensity $G/acT_{1}^{4}$ along the centerline (x=0.5) for (**a**) F1 scattering, (**b**) F2 scattering, (**c**) B1 scattering, (**d**) B2 scattering compared with the reference results.

The normalized net heat flux $Q_{y}/\left(acT_{1}^{4}/4\right)$ in the *y*-direction and radiation intensity $G/acT_{1}^{4}$ along the centerline (x=0.5) are compared with the reference results in [43]. Figure 5 and Figure 6 show the results for isotropic and different anisotropic phase function cases, respectively. It can be clearly observed that the computational results from our anisotropic-UGKP method align closely with the reference results for all cases. Furthermore, the influence of the anisotropic scattering on the energy transfer is also clearly shown: the forward scattering phase function transports more radiation heat than the isotropic case, which leads to a flatter distribution of radiation intensity; meanwhile, less radiation heat is transferred with the backward scattering phase function compared with the isotropic case, causing a steeper distribution of radiation intensity. These results verify the accuracy of the anisotropic-UGKP method in modeling 2D anisotropic-scattering models.

**Figure 7:**
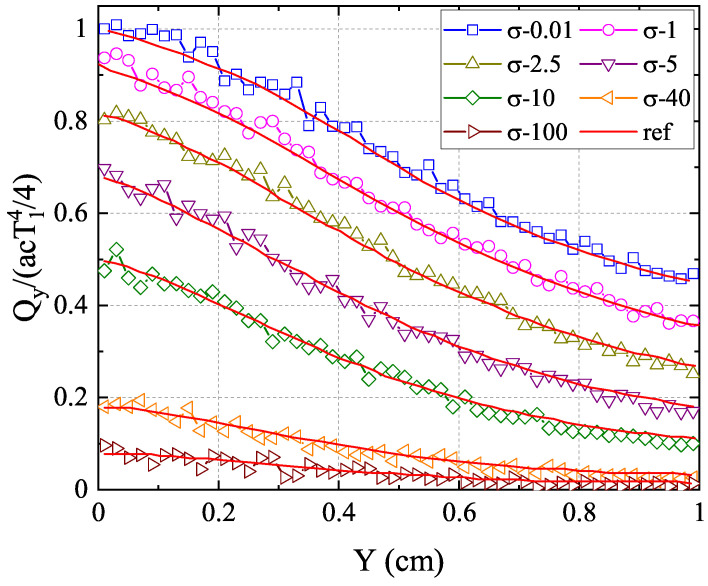
The normalized net heat flux $Q_{y}/\left(acT_{1}^{4}/4\right)$ in the *y*-direction along the centerline (x=0.5) with F2 scattering and different cross-sections compared with the reference results.

**Figure 8:**
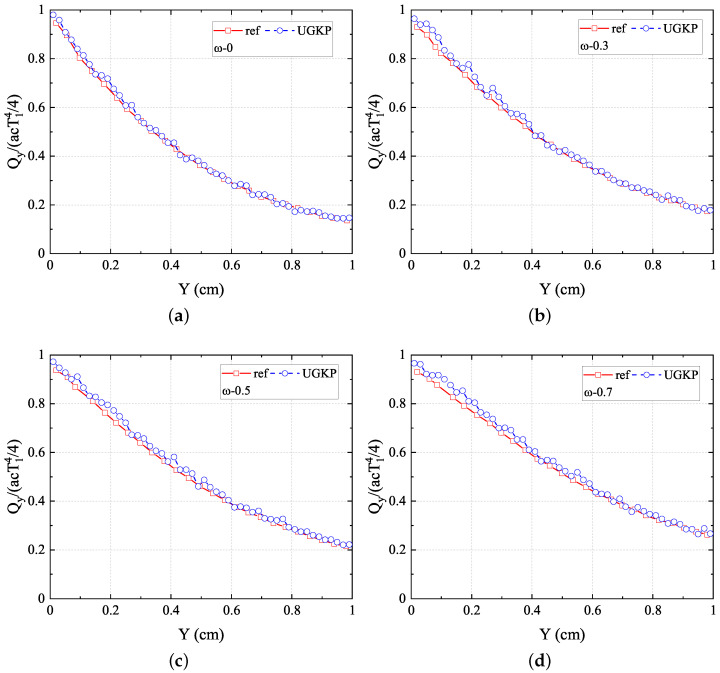
The normalized net heat flux $Q_{y}/\left(acT_{1}^{4}/4\right)$ in the *y*-direction along the centerline (x=0.5) for (**a**) ω=0 , (**b**) ω=0.3 , (**c**) ω=0.5 , (**d**) ω=0.7 compared with the reference results.

In addition, the F2 scattering case with varying cross-sections ranging from 0.01 to 100 is simulated. The results of the normalized net heat flux in the *y*-direction along the centerline (x=0.5) with different cross-sections are presented in Figure 7 compared with the reference results from [43] (σ=0.01 ∼ 10) and [44] (σ=40 ∼ 100) . As can be seen, the results of the anisotropic-UGKP method agree well with the reference results for all cases. Furthermore, Figure 8 displays the normalized net heat flux in the *y*-direction along the centerline (x=0.5) with different scattering albedos ω , which also shows good agreement with the reference results in [43]. With ω=0 , the energy absorption reduces the radiative heat transfer, which leads to the steepest distribution of $Q_{y}/\left(acT_{1}^{4}/4\right)$ . The absorption process becomes less important with ω increasing, causing a flatter distribution. The above results confirm the capability of the present anisotropic-UGKP method in simulating the absorbing and anisotropic scattering systems of all regimes.

### 3.3. Two-Dimensional (2D) Radiation Heat Transfer Problem with Collimated Incidence

**Figure 9:**
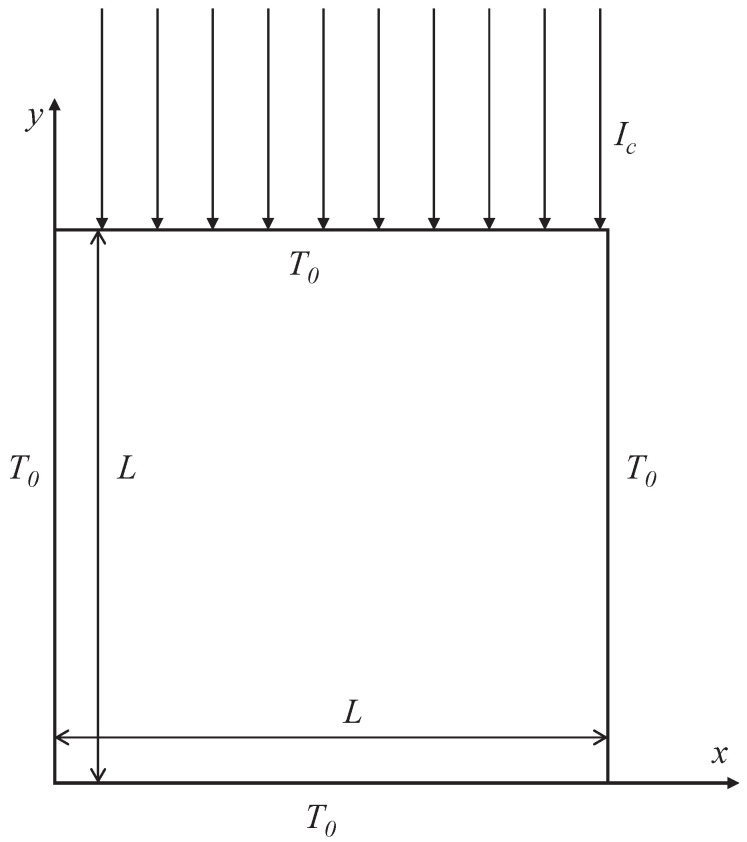
Schematic of 2D radiation heat transfer problem with collimated incidence.

We also take a 2D radiation heat transfer problem with collimated incidence to further test the present anisotropic-UGKP method. In this problem, the simulation domain is similar to the problem above. The difference lies in that the bottom wall is also kept cold, and a collimated beam is normally incident through the top wall with intensity $I_{c}$ , as illustrated in Figure 9. The scattering albedo is also set to be ω=1 for most of the cases considered here, and the same anisotropic phase functions are simulated.

**Figure 10:**
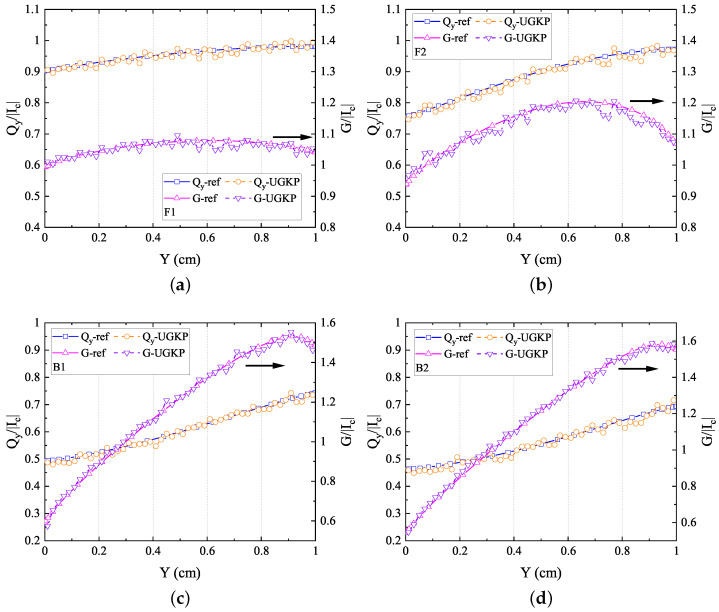
The normalized net heat flux $Q_{y}/\left|I_{c}\right|$ in the *y*-direction and radiation intensity $G/|I_{c}|$ along the centerline (x=0.5) for (**a**) F1 scattering, (**b**) F2 scattering, (**c**) B1 scattering, (**d**) B2 scattering compared with the reference results.

**Figure 11:**
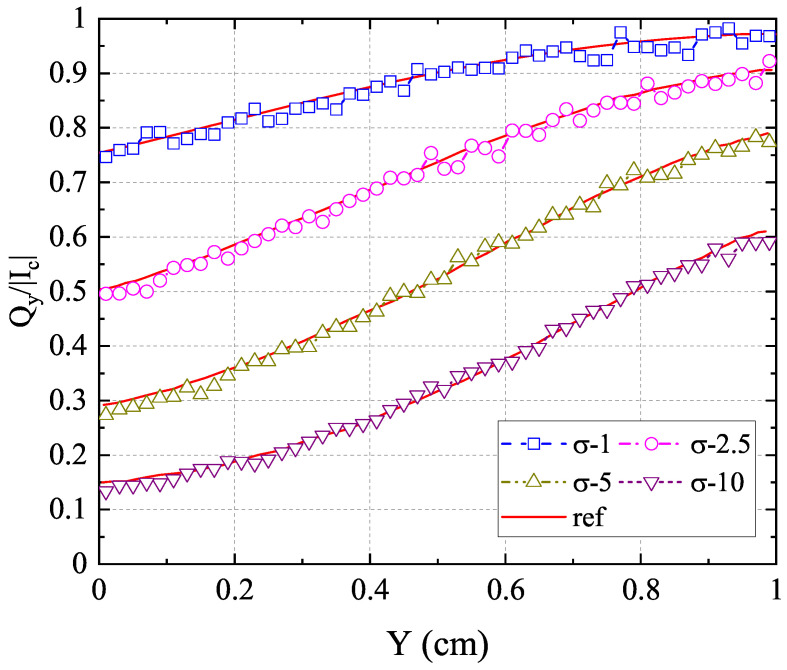
The normalized net heat flux $Q_{y}/\left|I_{c}\right|$ in the *y*-direction along the centerline (x=0.5) with F2 scattering and different cross-sections compared with the reference results.

**Figure 12:**
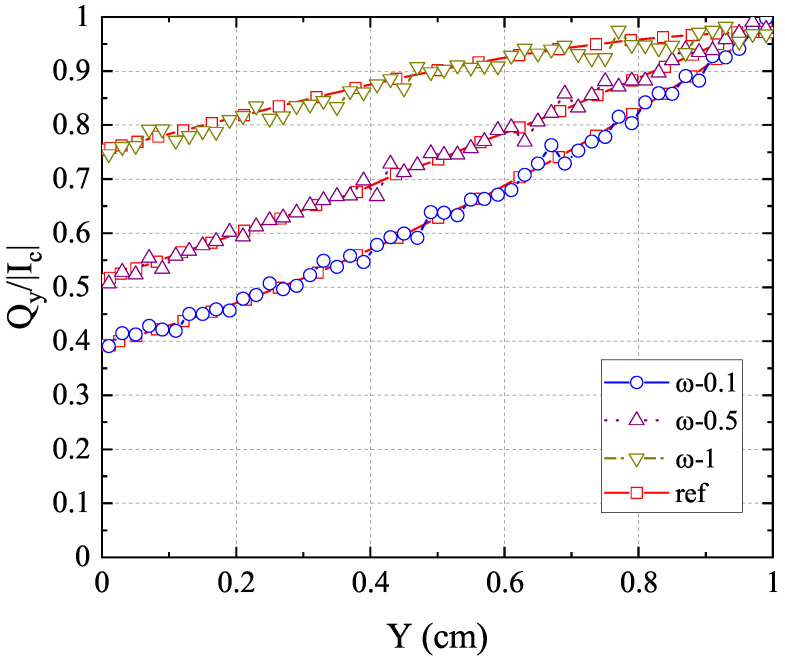
The normalized net heat flux $Q_{y}/\left|I_{c}\right|$ in the *y*-direction along the centerline (x=0.5) with F2 scattering and different albedos compared with the reference results.

The normalized net heat flux $Q_{y}/\left|I_{c}\right|$ in the *y*-direction and radiation intensity $G/|I_{c}|$ along the centerline (x=0.5) for different anisotropic phase functions are shown in Figure 10 compared with the reference results in [45]. The computational results from our anisotropic-UGKP method agree well with the reference results for all anisotropic cases. In addition, the normalized net heat flux values under different cross-sections and different albedos are given in Figure 11 and Figure 12, respectively. The results of the anisotropic-UGKP method also show good agreement with the reference results from [45]. Hence, the above results clearly show the capability of the present anisotropic-UGKP method for solving radiative transfer problems with absorbing and anisotropic scattering media in all regimes.

## 4. Conclusions

In this work, we developed the anisotropic-UGKP method based on our prior research. The macroscopic solver of the proposed anisotropic-UGKP method directly solves the macroscopic diffusion equations of the anisotropic scattering system. In addition, we proposed a revised multiscale phase function as an equivalent to the calculation and reconstruction of macroscopic scattering source *S*, covering from the free transport limit to the diffusion limit regimes. With this multiscale equivalent phase function, the scattered photon re-sampling process in the microscopic solver of the proposed anisotropic-UGKP method is simplified as one single scattering process. Therefore, the proposed anisotropic-UGKP method does not need to calculate the macroscopic fluxes and solve the macroscopic equations of high-order moments, significantly reducing computational costs. Furthermore, the anisotropic-UGKP method also has the asymptotic preserving (AP) property in both optically thin and thick regimes. A set of radiative transport problems, including 1D neutron problems and 2D radiation heat transfer problems, are conducted to validate the proposed anisotropic-UGKP method. All results agree well with the reference results from prior studies, showing the accuracy and efficiency of our anisotropic-UGKP method across a broad range of computational conditions. The scheme and code will be applied in the high-energy density physics engineering applications.

## Figures and Tables

**Table 1:** The Legendre expansion coefficients of the four anisotropic phase functions used in the 2D radiation heat transfer problem.

*l*	F1	F2	B1	B2
0	1.00000	1.00000	1.00000	1.00000
1	2.53602	2.00917	−0.56524	−1.20000
2	3.56549	1.56339	0.29783	0.50000
3	3.97976	0.67407	0.08571	
4	4.00292	0.22215	0.01003	
5	3.66401	0.04725	0.00063	
6	3.01601	0.00671		
7	2.23304	0.00068		
8	1.30251	0.00005		
9	0.53463			
10	0.20136			
11	0.05480			
12	0.01099			

## Data Availability

The data that support the findings of this study are available within the article.
